# Effectiveness of Blind & Ultrasound Guided Corticosteroid Injection in Impingement Syndrome

**DOI:** 10.5539/gjhs.v8n7p179

**Published:** 2015-11-18

**Authors:** Shila Haghighat, Parisa Taheri, Mohsen Banimehdi, Arash Taghavi

**Affiliations:** 1 Department of Physical Medicine and Rehabilitation, University of Isfahan Medical Sciences, Isfahan, Iran; 2 Department of Radiology, University of Isfahan Medical Sciences, Isfahan, Iran

**Keywords:** clinical outcome, corticosteroid, impingement syndrome, injection, ultrasound

## Abstract

Local steroid injections are common for treatment of impingement syndrome. Corticosteroid injections methods are basically in two formats, blind or with image guidance. The aim of this study is to compare the effect of ultrasound-guided in comparison with blind corticosteroid injections in patients with impingement syndrome. This study is a randomized clinical trial study undertaken in patients with diagnosis of impingement syndrome done in Isfahan University of Medical Science clinics from February 2014 to February 2015. The number of all patients registered in the study is 48; and then 40 patients were allocated to either control group randomly which received blind steroid injection or case group that underwent ultrasound-guided steroid injection. The clinical symptoms were assessed using Shoulder Pain and Disability Index (SPADI) questionnaire, Visual Analogue Scale (VAS) and shoulder range of motion (ROM) using goniometer at baseline and six weeks after the injection. Data analysis revealed a significant difference in the mean of the VAS, SPADI and shoulder ROM in both groups 6 weeks after intervention (P < 0.05). Patients with ultrasound guided corticosteroid injection had statistically significant improvements in function and shoulder ROM (abduction, flexion) compared to blind injection group after 6 weeks (P<0.05). There was not significant differences in pain (VAS) and internal and external rotation between these two groups (p>0.05). Our findings suggest that US image guided can improve the shoulder function of patients with impingement syndrome, and thus can be considered in comprehensive care programs of these patients for fast speed of rehabilitation.

## 1. Introduction

Painful shoulder problems are so common, as they are one of the main reasons of impaired function and statistically reported for 1 in 3 adult patients (Chard et al., 1991; Picavet et al., 2003), accounting for 1% of general practice (GP) consultations (Van Der Wind et al., 1995).

Subacromial impingement syndrome (SIS) is the most usual cause of shoulder pain with affecting one-half of all shoulder pain sufferers (Van Der Wind et al., 1995). SIS is generally observed with pain during rising of the arm, when a reduction in the space between the coracoacromial arch of the scapula and the humerus causes pinching of the subacromial/subdeltoid bursae, rotator cuff tendons and long head of biceps (Hanchard et al., 2004).

Many non-operative treatments have been advocated for treatment of subacromial impingement syndrome, including rest, active physiotherapy, non-steroidal anti-inflammatory drugs (NSAIDs), electrotherapy or cryotherapy (Brox et al., 1995). Corticosteroid injections are widely used to treat shoulder pain irrespective of the underlying etiology. The injections might be done in “blind” form which is via anatomical landmarks to guide placement a needle; or by using image guidance. Ultrasound (US) is a safe, effective imaging tool to guide musculoskeletal injections into the intended anatomical space (Skedros et al., 2007).

Studies have shown that the more accurate image guidance is than of needles placement (Hegedus, 2010). However, it is questionable whether the needle placement has a significant impact on clinical outcome. A recent RCT compared US -guided subacromial corticosteroid injection with systemic corticosteroid injection in patients with rotator cuff disease and found no difference in shoulder pain and function between groups (Ekeberg, et al., 2009). In 2011, one systematic review assessed Image-guided versus blind corticosteroid injections in adults with shoulder pain found that patients who underwent image-guided (ultrasound) injections had statistically significant greater improvement in shoulder pain and function at 6 weeks after injection (Roddy, 2014). These conflicting results may have come about from different study designs and evidence supports the need for a high quality trial to determine how to optimize treatments for SIS patients The main purpose of this study is to assess “in a standard clinic setting” whether US -guided subacromial corticosteroid injection provides better recoveries rather than unguided (blind) injection or not.

## 2. Material & Method

This study used a randomized clinical trial study method and was undertaken in Physical medicine and rehabilitation outpatient clinics in Isfahan University of Medical Science (Al-Zahra hospital) from February 2014 to February 2015. Patients were diagnosed based on clinical criteria, which were also confirmed by ultrasounds and x-rays for ruling out osseous pathology. 1) Posterolateral shoulder pain, 2) Pain in abduction or painful restriction of glenohumeral mobility, 3) provocation of symptoms by Neer and Hawkins tests.

Exclusion criteria were as follows: shoulder pain due to osseous pathology (e.g. osteoarthritis, osteonecrosis), duration of shoulder pain more than three months, previous trauma in the shoulder region, previous physiotherapy or local steroid injection in last three months, any evidence of predisposing condition such as diabetes mellitus, rheumatoid arthritis, hypothyroidism and unwillingness to participate in the present study(Roddy, 2014).

In total, 48 patients fulfilled the inclusion criteria of the study and were used for the study. Eight patients were excluded from the study due to predisposing conditions including diabetes (N=3), and unwillingness to participate in this study (N=5). Participants were randomly assigned to control and case groups, two groups of 20 subjects, using randomized allocation. In the each group patients received 40 mg methylprednisolone with 1cc lidocaine 2%. Posterior approach was used for subacromial bursa injection in anatomical (blind) method, In this way needle is placed approximately 2 cm below posterior-lateral of acromion and the needle guided forward and slightly to the left under the acromion(Frontra et al., 2008; Edmund & Wenyun, 2011).

In the second group corticosteroid injections were performed under ultrasound guidance. For subacromial bursal injection we used the lateral approach by using ultrasound equipment with high frequency linear transducer for guidance the probe is disinfected using the same disinfectant which is applied to the patient’s skin. The transducer was held in one hand and the syringe with 5 cm long needle caliber 23 Gauge in the other hand. The probe was positioned parallel to the long axis of the supraspinatus. The skin was punctured at a distance of about 2–3 cm from the probe in order to avoid contact between the needle and the probe. As soon as the needle had penetrated the subcutaneous tissues, its progress was real-time monitored on the US image. The needle was visualized as a hyperechoic structure with posterior comet-tail artifact. Progress of the needle until the tip had entered the subacromial bursa was observed. When the tip of the needle appeared to be inside the subacromial bursa, a small amount of liquid is injected to confirm the correct position (fluid passing from the needle tip into the bursa). The injection can be visualized in real-time as a spreading “cloud” of hyperechoic echoes inside the bursa (Molini et al., 2012).

The end points in the present study were changes in the Visual Analogue Scale (VAS) score for assessing pain severity (0 to 10), and the Shoulder Pain and Disability Index (SPADI), a scale for assessing pain and limitation of shoulder function from baseline and 6 weeks after intervention. The SPADI questionnaire instructed as a self-administered instrument, which its main purpose is to measure pain by using five items, and disability, using eight items, associated with shoulder complaints. The five items for pain included zero for “not any pain” and score 10 for “the worst pain”. Respectively, for the eight disability items, “no difficulty” received the score of zero and “difficulty requiring assistance” received the score of 10. The SPADI has good internal consistency, test-retest reliability, and criterion and construct validity. It can also detect change in patient status over time. Therefore, the SPADI can be a useful instrument both in clinical practice and in clinical research (Habermeyer et al., 2006). Shoulder range of motion was assessed using goniometer (according to the standard method).

This study had the approval of the ethics Committee of Vice Chancellor for Research of Isfahan University of Medical Sciences, projection no394037. All participants received trial information and provided written informed consent prior to participation in the research. In addition to that, the confidentiality of all information was strictly safeguarded by the researchers.

Study data were analyzed using the PASW Statistics 21 (IBM SPSS). Independent t-test carried out for compare the mean of Visual Analogue Scale (VAS), Shoulder Pain and Disability Index (SPADI) questionnaire, internal rotation and external rotation, flexion and abduction in two groups. Also, paired t-test was used to determine the effectiveness of USG injection and blindly injection in variables Visual Analogue Scale (VAS), Shoulder Pain and Disability Index (SPADI) questionnaire, internal rotation and external rotation, flexion and abduction at the first and six weeks after the injection. In all calculation P< 0.05 was consider as significant.

## 3. Results

This study used 40 patient participants, 20 patients in the blind injection group and 20 patients in the US-guided injection group. The participants were evaluated for the outcomes 6 weeks after the intervention.

The mean age of patients in US-guided injection group was 50.4±6.78 years with a range of 38 to 64 years, of whom 12 (60%) patients were female and 8 (40%) patients were male and mean body mass index (BMI) was 27.05±2.21. The blinded injection group consisted of 13 female (65%) and 7 (35%) male. The mean age of these patients was 52.3±7.48 years with a range of 41-65 years and mean BMI 26.03±3.11 recorded. No significant difference is observed in the demographic data between the two groups at the beginning of trial (P >0.05) indicating the success of randomization (Table 1).

**Table 1:** Demographic Characteristics of the Sample

Characteristic	Blind injection group (n =20) Mean ± SD			US guide injection group (n =20) Mean ± SD		P_1_-value^1^	P_2_-value
Age (years)	52.3±7.48			50.45±6.78		0.48	
BMI	26.35±3.83			27.05±2.21		0.41	
Duration (months)	1.87±0.48			1.8±0.54		0.64	

Gender		n	% of subjects	n	% of subjects		

	Male	7	35	8	40		0.74
	female	13	65	12	60		

1. P-values show the differences between the two groups. These are resulted from independent-sample t test.

The mean and standard deviation of Visual Analogue Scale (VAS), pain and disability scores of SPADI questionnaire, flexion, and abduction, internal, and external rotation are summarized in Table 2. Data analysis revealed a significant difference in the mean of Visual Analogue Scale (VAS), pain and disability score of the SPADI questionnaire, flexion, abduction, internal, and external rotation of shoulder 6 weeks after the intervention between the two groups (p<0.05) (Table 2).

**Table 2:** Comparing the mean ± SD of pain score, function and range of motion in patients with impingement syndrome at baseline and 6 weeks after following the injection of corticosteroids in two different methods, Based on SPADI^1^ & VAS^2^ questionnaires In patients referring to the Al-Zahra hospital clinic in 2014

Variable	Groups	Before intervention (Mean ± SD)	After intervention (Mean ± SD)	P1-value	Mean Differences (Mean ± SD	P_2_-value
VAS	US guide	8.1±1.11	3.25±1.16	<0.001	4.85±0.28	0.45
	Blind	6.75±1.16	2.3±1.78	<0.001	4.45±0.43

SPADI (pain)	US guide	37.65±6.5	14.05 ± 3.84	<0.001	23.6±1.45	0.34
	Blind	32.75±6.12	11.35±5.23	<0.001	21.4 ±1.76

SPADI (Disability)	US guide	53.45± 9.94	22.35± 5.55	<0.001	31.1± 2.2	0.01
	Blind	42.1± 10.41	18.95±7.19	<0.001	23.15± 2.32

Abduction	US guide	132.5±17.5	146.25±18.76	<0.001	13.75±2.98	0.01
	Blind	129±21.67	136.25±13.06	0.04	7.25±3.45

Flexion	US guide	147.5±15.43	156.75±13.4	<0.001	9.25±1.9	0.04
	Blind	145.25±9.93	150±8.88	0.03	4.75±2.06

External rotation	US guide	68±12.07	72±10.8	0.004	4±1.23	0.92
	Blind	71.5±16.94	75.75±17.49	0.04	4.25±2.21

Internal rotation	US guide	62.25±16.42	67.25±15.25	0.002	5±1.35	0.73
	Blind	62±17.27	67.75±12.92	0.003	5.75±1.71

1 Shoulder Pain And Disability Index.

2 Visual Analogue Scale.

P_1_ values show the differences after and before the study in each group and are resulted from paired sample t test.

P_2_ values show the differences between the two groups and are resulted from independent sample t test.

In comparison of between two groups, US guided group experienced significantly better improvement in disability score of the SPADI questionnaire (p=0.01), abduction (p=0.01) and flexion (p=0.04), and significant differences was not seen in pain (VAS) (p=0.45), pain scores of SPADI questionnaire (p=0.34), internal rotation (p=0.73) and external rotation (P=0.92) between the two groups (Table 2).

## 4. Discussion

Musculoskeletal Ultrasound has become so common as the result of improved technology and better resolution to guide environmental joints and soft tissue injection. US has the unique advantage of being able to visualize soft tissues, bony land-marks, and the needle with real-time scanning, thereby allowing dynamic visualization (Braddom, 2010). Although the US image guidance has been shown to enhance the accuracy of injections, it has not been proven that enhanced accuracy is related to an enhanced efficacy (Hall & Buchbinder, 2004).

The aim of this study was to assess the efficacy of Ultrasound -guided versus blind corticosteroid injections in improving pain and function in patients with impingement syndrome. Our results showed that patients received ultrasound -guided steroid injection had significantly better improvement in function. Among ROM, shoulder flexion and abduction showed improvement in the US-guided group compared to the control group.

US-guided corticosteroid injection had superior efficacy when compare to blind injection at least in shoulder function. This result was confirmed by shoulder flexion and abduction evaluation. However, US-guided corticosteroid injection was as effective as blind injection in respect to shoulder pain and other ROM parameter.

In 2004, Naerdo et al studied 41 patients with painful shoulder. Patients were randomized to receive a blind subacromial injection (n = 20) or a sonographic guided injection (n = 21). Six weeks after injection, the VAS and the SFA (Shoulder function assessment) score showed a significantly greater improvement in sonographic guided injection group compared with blind group (Naredo et al., 2004).

In study of Ucuncu et al, in 2009 a total 60 consecutive patients with shoulder pain was enrolled, and randomly assigned to either by landmark-guided (n=30) or USG guided (n=30) injection. Clinical assessment included VAS for pain, the Constant scale for function, passive and active shoulder ROM with goniometric evaluation. Six weeks later the VAS and the Constant score showed a significant improvement in USG group compared with landmark-guided group. There were significant improvements in both active and passive ROM (Ucuncu et al., 2009).

On the other hand, there was some evidence that did not demonstrate benefits in the US–guided steroid injection group compared to the control group (Hegedus et al., 2010). In 2012, Bloom et al in a systematic review by survey of 5 trials that compared efficacy of corticosteroid USG injection with blind injection concluded that ultrasound only improves injection accuracy (Bloom et al., 2010). Dogu et al. (2013) performed a study on forty-six patients with subacromial impingement syndrome for ultrasonography-guided and blind corticosteroid injections. All patients were assessed before the injection and 6 weeks after the injection. After sixth week ending, all patients showed some improvements in all parameters including pain, function, & ROM, whether the injected mixture was found or not.

Other studies suggested that possible therapeutic mechanisms of local steroid injections on impingement syndrome include anti-inflammatory effects, relaxation of reflex muscle spasm, pain relief, mechanical improvement and the placebo effect (Neustadt et al, 1991).

The small sample size of study would not allow elaboration of the efficacy of ultrasound guided injection. To determine long-term effects of the technique and also to compare ultrasound guided steroid injection to other conservative management of impingement syndrome further research is needed.

## 5. Conclusion

The results of this study shows that US-image guided method can improve the overall shoulder function of patients with impingement syndrome, thus, it can be suggested to special programs for fast speed rehabilitation of these patients.

We can list the advantages of this study as following:

1) This study has been done in a radiologist clinic with the help of an experienced sonography soft texture.
2) There is no similar study in the studies population.
3) Results of this study show that, which are in line with other studies, that ultrasound-guided injection increases accuracy but it does not have a significant impact on efficacy, especially in cases where there are limitations to ultrasound.
